# Influence of Fish Oil-Derived n-3 Fatty Acid Supplementation on Changes in Body Composition and Muscle Strength During Short-Term Weight Loss in Resistance-Trained Men

**DOI:** 10.3389/fnut.2019.00102

**Published:** 2019-07-16

**Authors:** Jordan D. Philpott, Niels J. Bootsma, Nidia Rodriguez-Sanchez, David Lee Hamilton, Elizabeth MacKinlay, James Dick, Samuel Mettler, Stuart D. R. Galloway, Kevin D. Tipton, Oliver C. Witard

**Affiliations:** ^1^Physiology, Exercise and Nutrition Research Group, Faculty of Health Sciences and Sport, University of Stirling, Stirling, United Kingdom; ^2^Institute for Physical Activity and Nutrition (IPAN), School of Exercise and Nutrition Sciences, Deakin University, Geelong, VIC, Australia; ^3^Institute of Aquaculture, University of Stirling, Stirling, United Kingdom; ^4^Department of Health Sciences and Technology, ETH Zurich, Zurich, Switzerland; ^5^Department of Sport and Exercise Sciences, Faculty of Social Sciences and Health, Durham University, Durham, United Kingdom; ^6^Centre for Human and Applied Physiological Sciences, School of Basic and Medical Biosciences, Faculty of Life Sciences and Medicine, King's College London, London, United Kingdom

**Keywords:** omega-3 polyunsaturated fatty acids, energy restriction, fat-free mass, fat mass, performance, athletes

## Abstract

**Background:** A detrimental consequence of diet-induced weight loss, common in athletes who participate in weight cutting sports, is muscle loss. Dietary omega-3 polyunsaturated fatty acids (n-3PUFA) exhibit a protective effect on the loss of muscle tissue during catabolic situations such as injury-simulated leg immobilization. This study aimed to investigate the influence of dietary n-3PUFA supplementation on changes in body composition and muscle strength following short-term diet-induced weight loss in resistance-trained men.

**Methods:** Twenty resistance-trained young (23 ± 1 years) men were randomly assigned to a fish oil group that supplemented their diet with 4 g n-3PUFA, 18 g carbohydrate, and 5 g protein (FO) or placebo group containing an equivalent carbohydrate and protein content (CON) over a 6 week period. During weeks 1–3, participants continued their habitual diet. During week 4, participants received all food items to control energy balance and a macronutrient composition of 50% carbohydrate, 35% fat, and 15% protein. During weeks 5 and 6, participants were fed an energy-restricted diet equivalent to 60% habitual energy intake. Body composition and strength were measured during weeks 1, 4, and 6.

**Results:** The decline in total body mass (FO = −3.0 ± 0.3 kg, CON = −2.6 ± 0.3 kg), fat free mass (FO = −1.4 ± 0.3 kg, CON = −1.2 ± 0.3 kg) and fat mass (FO = −1.4 ± 0.2 kg, CON = −1.3 ± 0.3 kg) following energy restriction was similar between groups (all *p* > 0.05; d: 0.16–0.39). Non-dominant leg extension 1 RM increased (6.1 ± 3.4%) following energy restriction in FO (*p* < 0.05, *d* = 0.29), with no changes observed in CON (*p* > 0.05, *d* = 0.05). Dominant leg extension 1 RM tended to increase following energy restriction in FO (*p* = 0.09, *d* = 0.29), with no changes in CON (*p* > 0.05, *d* = 0.06). Changes in leg press 1 RM, maximum voluntary contraction and muscular endurance following energy restriction were similar between groups (*p* > 0.05, *d* = 0.05).

**Conclusion:** Any possible improvements in muscle strength during short-term weight loss with n-3PUFA supplementation are not related to the modulation of FFM in resistance-trained men.

## Introduction

The application of diet-induced weight loss extends beyond clinical (overweight and obese) populations. Athletic populations competing in weight-category sports (e.g., boxing), or sports where a high power-to-body mass ratio (sprinting) or aesthetics (gymnastics) are pre-requisites for success also routinely periodize their training programme to include short-term periods of energy restriction (1). However, a counterproductive feature of diet-induced weight loss in athletes that accompanies the reduction in fat mass includes the decline in fat-free mass (FFM), specifically of skeletal muscle tissue (2, 3).

Changes in body composition during diet-induced weight loss can be manipulated with nutrition (4). Most notably, experimental studies demonstrate that increasing dietary protein intake confers an effective nutritional strategy to promote high-quality weight loss during energy restriction, i.e., loss of fat mass while maintaining muscle mass during short-term weight loss (2, 5). However, the preservation of muscle mass during energy restriction with a higher protein intake did not translate into the better maintenance of exercise performance in resistance-trained young men (2). The importance of other nutrients for maintaining FFM during diet-induced weight loss has been proposed (2, 5), but few experimental studies have addressed the effectiveness of these nutrients on changes in body composition during weight loss.

Another potentially effective nutritional intervention to promote high-quality weight loss during energy restriction in athletic populations is the ingestion of omega-3 polyunsaturated fatty acid (n-3PUFA). Both *in vitro* cell line experiments (6, 7) and *in vivo* human studies (8–10) support the notion that n-3PUFA exhibit anabolic properties, in particular the omega-3 species eicosapentaenoic acid (EPA). Previous proof-of-principle studies have demonstrated that fish oil-derived n-3PUFA supplementation potentiated the response of muscle protein synthesis (MPS) to the infusion of amino acids and insulin (9, 10), and enhanced muscle mass (11) and strength (12, 13) in older adults. The mechanism most commonly proposed to underpin the anabolic action of n-3PUFA relates to modifying the lipid profile of the muscle phospholipid membrane. This structural change in integrity of the muscle membrane is understood to activate intracellular signaling proteins (e.g., mTORC1-p70S6k1) (9, 14) that upregulate muscle protein synthesis (MPS), thus modulating muscle mass.

Based on current evidence from experimental studies, the metabolic role of n-3PUFA in regulating muscle mass is most evident under catabolic conditions. Consistent with this notion, fish oil-derived n-3PUFA supplementation was shown to exhibit protective roles in preserving muscle mass in a clinical population of cancer cachexia patients (15) and following a short-term period of leg immobilization in healthy recreationally-active young women (8). Another catabolic situation is diet-induced weight loss, whereby the intracellular activation of AMPK signals an energy deficit within the muscle cell (16). Given that MPS is an energetically expensive process, requiring ~4 moles of ATP to bind each amino acid during the elongation process of translation (17), this activation of AMPK acts to conserve energy during weight loss by down-regulating basal rates of MPS (16, 18, 19). To our knowledge, all studies to date that have investigated the impact of fish oil supplementation on body composition during weight loss have been conducted in a clinical setting with overweight and/or obese patients (20, 21). Given the link between dietary n-3PUFA intake, MPS and muscle mass during injury-simulated leg immobilization in trained young women (8), there is strong rationale to support a protective role of n-3PUFA in maintaining muscle mass and strength during energy-restricted weight loss in athletes.

The primary aim of this study was to investigate the influence of fish oil-derived n-3PUFA supplementation during short-term diet-induced weight loss on changes in body composition and muscle strength in resistance-trained young men. We hypothesized that n-3PUFA supplementation would attenuate the loss of lean body mass and better maintain lower limb muscle strength following a 2 week period of an energy-restricted diet compared to placebo.

## Methods

### Study Design

**Figure 1:**
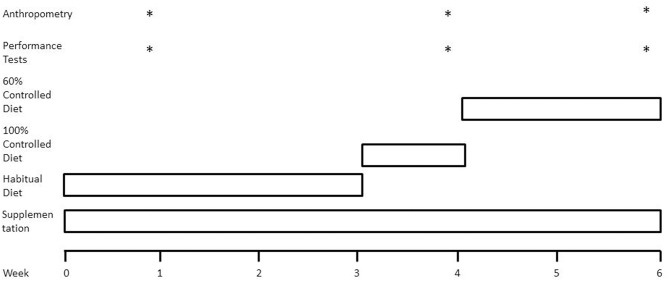
Schematic overview of study design. Weeks 0–3, participants consumed their habitual diet under free-living conditions. Week 4, all food items were supplied by researchers to ensure participants consumed a diet constituting 100% of habitual energy intake and a macronutrient composition of 50% carbohydrate, 35% fat, and 15% protein. Weeks 5 and 6, all food items provided to ensure energy-restricted diet was equivalent to 60% of habitual energy intake. *Represents when the category occurs displayed in the figure.

Using a parallel research design adapted from Mettler et al. (2) (Figure 1), participants were randomly assigned to one of two groups: a fish oil supplement group (FO) or an energy and macronutrient matched control group (CON). Participants consumed their assigned supplement twice daily for the entire 6-week study period. Participants consumed their habitual diet for the first 3 weeks of the study, with week 1 used to assess energy intake and energy expenditure. During week 4, all food items and fluids were supplied by researchers, providing 100% of habitual energy intake. During weeks 5 and 6, the energy content of the diet was reduced to 60% of habitual intake. At the end of weeks 1, 4, and 6, measurements of body mass, body composition, and muscle performance were obtained under controlled laboratory conditions using dual energy x-ray absorptiometry (DXA), leg extension and leg press fixed resistance machines (Cybex International, Illinois, USA) and isokinetic dynamometry technology.

### Participant Recruitment and Ethical Approval

Twenty healthy (no known metabolic disorders as determined by health questionnaire) young resistance-trained males were recruited from local sports clubs. All participants had undertaken resistance training for at least the previous 6 months, were currently training ≥2 times per week, and were not consuming supplements containing n-3PUFA at the time of study enrolment. Participants were asked to continue their habitual training throughout the 6-week study period. The West of Scotland Research Ethics Service approved the study procedures.

### Dietary Control and Supplementation

In a single blinded fashion, participants were divided equally between supplement conditions, consuming 2 × 200 mL volume juice-based drinks (1 × morning and 1 × evening) daily over the 6 week supplementation period (Smartfish Sports Nutrition, Ltd). Drinks were provided to match energy and macronutrient composition of the two diets. However, the FO beverage contained an additional 2 g of n-3PUFA per drink. Supplements were taste-matched and equal in protein and carbohydrate content. The experimental supplement condition contained 2 g of fish oil (~1 g of EPA and ~1 g of DHA) whereas the placebo condition did not contain fish oil. The additional energy provided by the fish oil supplement in the experimental condition was accounted for by modifying the energy content of the background diet.

### Trial Days and Measurements

Testing sessions commenced at ~07:00 on weeks 1 (day 7), 4 (day 27), and 6 (day 41) following an overnight fast and having consumed 500 ml of water 1–2 h prior to arriving at the laboratory. Participants were instructed to empty their bladder before body weight was measured using standard laboratory scales (Seca Quadra 808, Birmingham, UK) with participants wearing underwear only. Body composition was measured using a narrowed fan-beamed dual-energy x-ray absorptiometry (iDXA GE Healthcare) with analysis performed using GE Encore 13.40.038 Software (GE Healthcare). All DXA scans followed procedures previously described by Rodriguez-Sanchez and Galloway (22) and were performed by the same trained technician.

### Muscle Strength and Endurance

The first test of muscle strength was a single leg isokinetic/eccentric maximum voluntary contraction (MVC) of the knee flexors using an isokinetic dynamometer (Biodex Corporation, New York). Participants were seated on the dynamometer with their upper body, hips, and thigh securely strapped into the seat and the hip at a 90° angle to the legs. The lower leg was attached to the arm of the dynamometer 1 cm above the lateral malleolus ankle joint with the axis of rotation of the dynamometer arm aligned with the lateral femoral condyle. The dynamometer arm was set to start and stop at angles 90° and 0°, respectively, at the knee joint. Participants were asked to use maximum effort resist the dynamometer arm from moving the knee joint from a 90° to a 0° angle. Each participant performed 3 ×3 sets/reps of this MVC protocol with a 60 s rest between sets. Each participant's greatest peak torque from the three sets was recorded.

Following 5 min rest, unilateral 1 RM for leg extension was assessed using a previously validated protocol (23) on a fixed resistance machine (Cybex International Inc, Cybex International, MA). Seat and knee position was recorded during testing session and was replicated during weeks 4 and 6. On the same day, following a 10 min rest period, unilateral muscular endurance was measured. Participants completed as many repetitions as possible on leg extension and leg press, with resistance set at 60% of individual baseline 1 RM. Participants completed repetitions at their own pace but were instructed to cease exercising as soon as a rest between repetitions was required. In total, testing sessions were completed within 180 min.

### Diet

During weeks 1–3, all participants consumed their habitual diet but were asked to refrain from eating oily fish to ensure that supplementation accounted for changes in blood lipid profiles. During week 1, energy intake and energy expenditure were measured. Energy intake was measured using a 3-d food report. On the same days as the food report, energy expenditure was measured using the physical activity questionnaire (24) and from Actiheart data (CamNtech Ltd, Papworth Everard, England). All measures of energy intake and expenditure were averaged to give a 100% energy value. During week 4, participants were instructed to consume only the food provided by researchers that contained 100% of their habitual diet with a macronutrient composition of 50% carbohydrate, 35% fat, and 15% protein. The energy content of the supplement was taken into account when calculating the habitual energy intake of each participant. The only exceptions were water and diet soft drinks that could be consumed *ad libitum*. Participants were asked to provide feedback on the volume of food consumed. If a participant reported feeling hungry, the energy content of the diet was increased. Conversely, if the volunteer was unable to eat all food provided, the energy content of the diet was reduced. Participants also were asked to monitor their body weight throughout week 4 to ensure body weight stability. During weeks 5 and 6, dietary energy intake was reduced to 60% of habitual intake, however macronutrient composition remained constant. Throughout weeks 4–6, foods rich in omega-3 fatty acids such as oily fish (tuna, salmon, mackerel), walnuts and margarine were omitted from the diet. The diets were individually tailored to compensate for individual eating patterns and preferences and therefore maximize diet compliance. During weeks 4–6, participants were asked to return any food that was not consumed to researchers for weighing. Returned food was then weighed and an energy content was calculated. The energy content not consumed by the participant was added on to the following day's diet. Every attempt was made to provide participants with the confidence to honestly report any non-compliance without any consequences.

### Blood Analysis

Approximately 1 mL of venous blood was dispensed onto specialized Whatman 903 blood collection cards (GE Healthcare Ltd, Forest Farm Industrial Estate, Cardiff, CF 14 7YT, UK). Cards were dried for 3 h after which the dried whole blood sample was detached from the collection device using forceps and placed into a screw-cap vial containing 1 mL of methylating solution (1.25 M methanol/HCl). Vials were then placed in a hot block at 70°C for 1 h. The vials were allowed to cool to room temperature before adding 2 mL of distilled water and 2 mL of saturated KCl solution. Fatty acid methyl esters (FAME) were then extracted using 1 ×2 mL of isohexane + BHT followed by a second extraction using 2 mL of isohexane alone. This extraction method has been previously validated as a reliable measure of whole blood fatty acid composition in our own laboratories (25). FAME were then separated and quantified by gas liquid chromatography (ThermoFisher Trace, Hemel Hempstead, England) using a 60 m ×0.32 mm ×0.25 μm film thickness capillary column (ZB Wax, Phenomenex, Macclesfield, UK). Hydrogen was used as carrier gas at a flow rate of 4.0 mL·min−1 and the temperature program was from 50 to 150°C at 40°C·min−1 then to 195°C at 2°C·min−1 and finally to 215°C at 0.5°C·min−1. Individual FAME were identified compared to well-characterized in house standards as well as commercial FAME mixtures (Supelco™ 37 FAME mix, Sigma- Aldrich Ltd., Gillingham, England).

### Data Presentation and Statistical Analysis

Data were analyzed using Statistical Package for Social Sciences 21 (IBM SPSS, Chicago, IL). All data were found to be normally distributed based on the Shapiro-Wilk test. Differences across time for body composition, muscle strength and muscular endurance were analyzed by a mixed-design, two-way (time and supplement group) ANOVA. Two between-subject variables (FO and PLA) and either three within-subject (weeks 1 (habitual diet), 4 (100% diet), and 6 (60% diet) time-points) or 2 within-subjects (week 4 and week 6) variables were modeled within the two-way ANOVA. Where a significant time × supplement group interaction was detected, a Bonferroni *post hoc* test was performed to locate the timepoint(s) whereby differences existed between supplement groups. Statistical significance was set at the level of ≤ 0.05. Cohen's effect sizes (d) were calculated to compare differences between conditions. Effect sizes of 0.2 were considered small, 0.5 considered medium, and >0.8 were considered large (26). All data were expressed as means ± SD, unless otherwise stated.

## Results

### Supplement Control

All participants consumed all of the supplements provided to them. No adverse events occurred due to the fish oil or placebo supplementation.

### Dietary Intake During Energy Restriction

**Figure 2:**
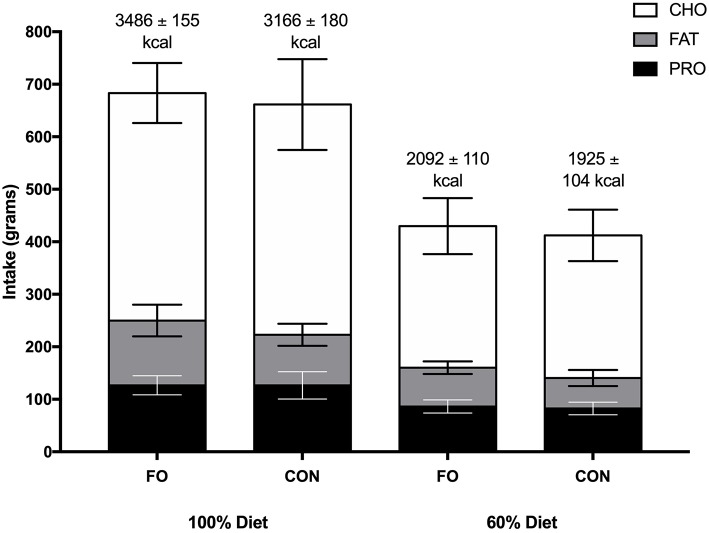
Energy intake (kcal) and macronutrient composition (grams) during the 100% diet period and the 60% diet period in fish oil (FO) and control (CON) supplement groups. Values are means ± SD. CHO, carbohydrate; PRO, protein.

No differences in energy or macronutrient intakes were observed between FO and CON during the habitual diet, 100% diet, and 60% diet periods (*p* > 0.05, Figure 2). Energy intake was lower during the 60% diet period compared to the 100% diet period in both conditions (*p* < 0.001).

### Blood n-3PUFA Composition

**Figure 3:**
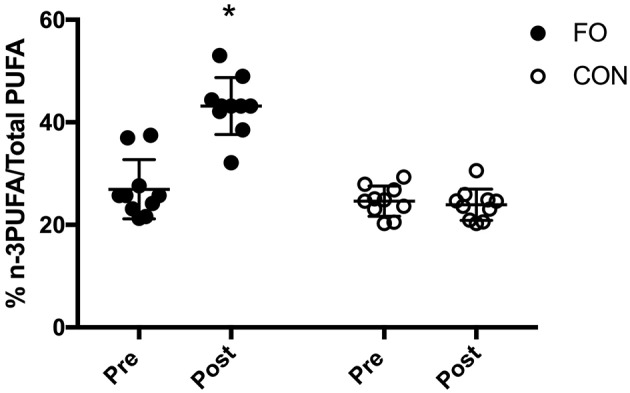
Blood % n-3PUFA/total PUFA composition before (Pre) and after (Post) 6 week of supplementation. Data are expressed as means ± SD and also as individual values. *Significant difference vs. baseline (Pre) in corresponding supplement group.

Baseline (pre) blood % n-3PUFA/totalPUFA composition was similar between groups (*p* < 0.01, Figure 3). Blood n-3PUFA composition increased by ~60% following 6 weeks of supplementation in FO, whereas no change was observed in CON. At the individual level, blood % n-3PUFA/totalPUFA composition increased in all 10 participants after supplementation in FO.

### Body Composition

**Figure 4:**
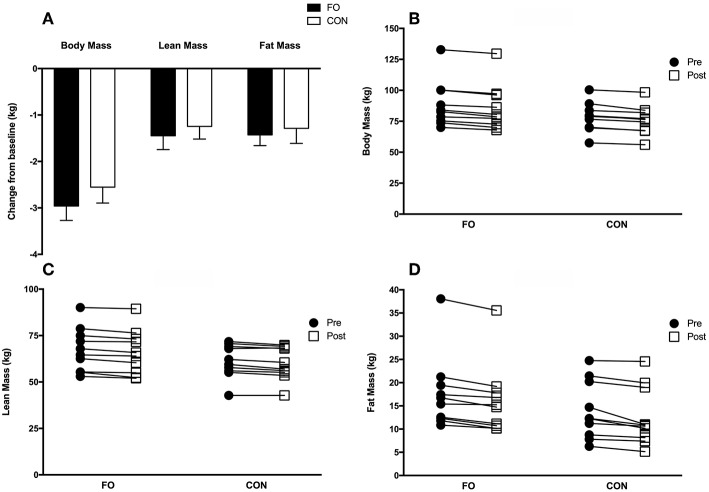
Group **(A)** and individualized changes in total body mass **(B)**, lean body mass **(C)**, and fat mass **(D)** from baseline (average of the two measurements collected during week 1 (Habitual diet) and 4 (100% diet) prior to weight loss) following 2 weeks of 40% energy restriction in fish oil (FO) and control (CON) supplement groups. Values are means ± SEM.

Total body mass (pre: 83.6 ± 3.6 kg; post: 80.8 ± 3.5 kg, *p* < 0.001), lean body mass (pre: 64.4 ± 2.3 kg; post: 63.0 ± 2.3 kg, *p* < 0.001), and fat mass (pre: 15.8 ± 1.6 kg; post: 14.4 ± 1.6 kg, *p* < 0.001) for all participants, decreased from baseline (pre) following 2 weeks of energy restriction (Figure 4A), with no differences between conditions. Individual changes in body mass, lean body mass and fat mass are presented in Figures 4B–D. Regional changes in total body mass, lean body mass, and fat mass were similar between FO and CON (see Supplementary Table).

### Muscle Strength

**Figure 5:**
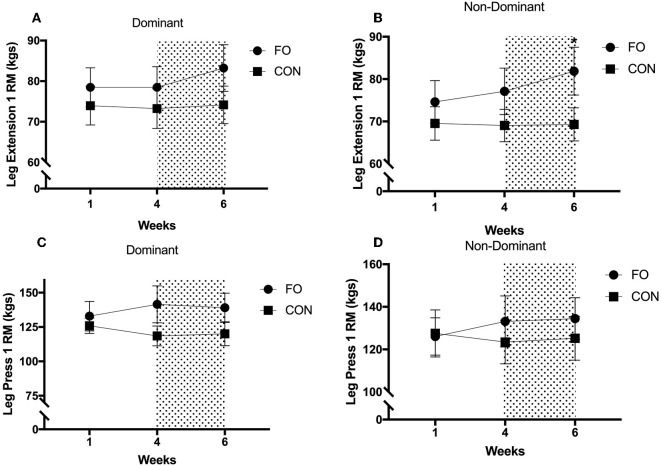
One repetition maximum for **(A)** dominant leg extension, **(B)** non-dominant leg extension, **(C)** dominant leg press, and **(D)** non-dominant leg press during weeks 1 (habitual diet), 4 (100% diet), and 6 (60% diet) of study. Shaded area represents the 2 week period of energy restriction. Values are means ± SEM. *Significant difference compared to weeks 1 and 4 in corresponding supplement group (*p* < 0.05).

Leg press and leg extension 1RM remained constant between week 1 and week 4 for both dominant and non-dominant legs (all *p* > 0.30, Figure 5). Leg extension 1 RM for the non-dominant leg increased by 6.1 ± 3.4% following energy restriction (weeks 4–6) compared with energy balance (weeks 0–4) in FO (*p* < 0.05, *d* = 0.29), with no changes in CON across the 6 week period. Leg extension 1 RM for the dominant leg tended to increase following energy restriction in FO (*p* = 0.092, *d* = 0.29), whereas no changes were observed in CON. No differences in leg press 1 RM for either leg were observed between weeks 4 (pre 100% diet period) and 6 (post 60% diet period) in either supplement group.

**Figure 6:**
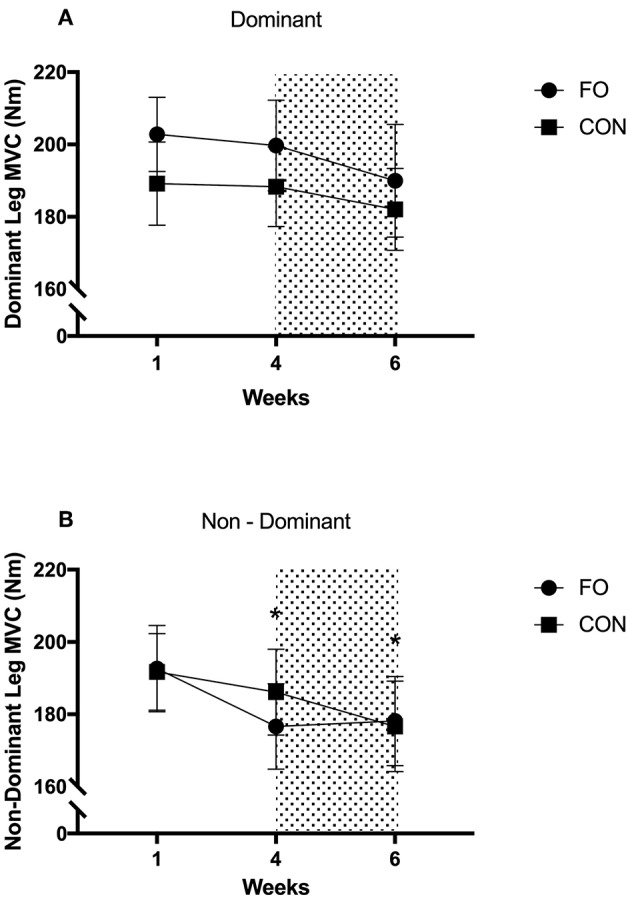
Maximum Voluntary Contraction of dominant and non-dominant legs during week 1 (habitual diet), week 4 (100% diet), and week 6 (60% diet). Shaded area represents 2 week period of energy restriction. Values are mean ± SEM. **(A)** dominant leg. **(B)** non-dominant leg. *Significant difference compared to week 1.

There were no differences in MVC for the dominant leg across diet periods or between supplement groups (Figure 6). MVC for the non-dominant leg decreased by 5.7 ± 7.9% from week 1 to 4 (*p* = 0.03, *d* = 0.31) and by 7.4 ± 11.8% from week 1 to 6 (*p* = 0.016, *d* = 0.42), with no differences between supplement groups.

### Muscular Endurance

**Figure 7:**
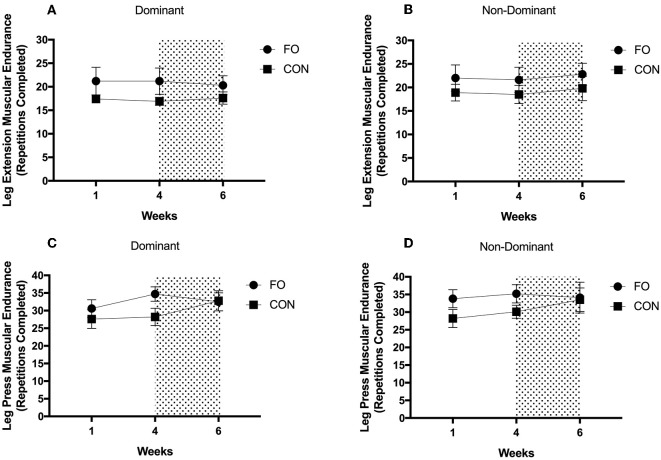
Muscular endurance for **(A)** leg extension on dominant leg, **(B)** leg extension on non-dominant leg, **(C)** leg press on dominant leg, and **(D)** leg press on non-dominant leg during weeks 1 (habitual diet), 4 (100% diet), and 6 (60% diet) of study protocol. Shaded area represents a 2 week period of energy restriction. Values are means ± SEM.

There were no differences in muscular endurance across diet periods or between supplement groups in either the dominant or non-dominant leg for leg extension or leg press exercises (all *p* > 0.05, Figure 7).

## Discussion

The primary aim of this study was to investigate the influence of dietary fish oil-derived n-3PUFA supplementation on changes in body composition and muscle strength during a short-term period of weight loss in resistance-trained young men. Our findings indicate that n-3PUFA supplementation resulted in a partial improvement in muscle strength following 2 weeks of 40% energy restriction, i.e., a small improvement in 1 RM leg extension in FO, but changes in MVC we similar between FO and CON groups. However, refuting our original hypothesis, n-3PUFA supplementation failed to modulate changes in body composition or attenuate the decline in muscle endurance induced by short-term weight loss. The practical implications of these preliminary data remain unclear, but suggest that dietary n-3PUFA supplementation may maintain, if not improve, some components of muscle strength during short-term weight loss in athletes competing in weight category sports and/or sports that depend on a high power-to-body mass ratio. However, the causal mechanism(s) that underpin this muscle adaptive response does not appear to be related to the modulation of lean body mass.

Changes in muscle strength induced by diet and exercise training are often associated with changes in muscle mass. Despite the improvement in 1 RM leg extension with n-3PUFA supplementation following the 2 week period of diet-induced weight loss, the decline in fat-free mass was comparable between supplement groups. Our laboratory previously demonstrated that 4 weeks of fish oil supplementation markedly increased n-3PUFA concentrations in the muscle cell (14). The uptake of n-3PUFA into the muscle cell membrane has been suggested to prime the muscle translational machinery inside the cell to respond to anabolic stimuli in both young (10) and older (9) adults. Moreover, a recent study demonstrated that fish oil supplementation attenuated the decline in muscle mass following 2 weeks of limb immobilization in trained young women, as mediated by a greater integrated response of MPS (8). Given that the primary locus of control for the regulation of muscle mass in resistance-trained young men is MPS (27), we hypothesized that any improvement in muscle strength during weight loss with n-3PUFA supplementation would be mediated by the preservation of lean body mass. However, in the present study, non-dominant leg extension 1 RM increased by >6% with n-3PUFA supplementation following 2 week of diet-induced weight loss, despite a 1.4 kg decrease in FFM. This apparent disconnect between muscle strength and FFM is not uncommon (28) and cannot be explained from this proof-of-principle study. Nonetheless, even if the incorporation of n-3PUFA into the phospholipid layer of the muscle cell membrane led to an upregulation of the muscle protein synthetic machinery, it did not appear to mediate the improvement in muscle strength observed following diet-induced weight loss in the n-3PUFA group.

A feasible alternative explanation for the improvement in 1 RM leg extension following energy restriction with n-3PUFA supplementation, in the absence of any changes in lean body mass, may relate to neuromuscular function. Consistent with this notion, a previous study demonstrated a reduction in electro-mechanical delay, defined as the time taken for a specific muscle to respond to a stimulus, with n-3PUFA supplementation, albeit in older adult women (12). From a mechanistic standpoint, DHA is an essential component of the phospholipid membrane of neurons localized in brain tissue (29). Moreover, previous animal and human studies have reported strong associations between increased DHA concentrations in brain tissue (30), improvements in brain function and increased muscle strength (31). In the present study, we report a marked increase in blood n-3PUFA concentrations following 4 weeks of n-3PUFA supplementation containing 2 g of DHA per day. On the basis of comparable findings using a rodent model (32), it is reasonable to assume that DHA concentrations also increased in the neural tissue of our resistance-trained men. Although speculative, these data provide preliminary support for the notion that neuromuscular mechanisms may underpin the current observation of a maintenance, if not increase, in muscle strength following an energy-restricted diet in the n-3PUFA group.

The divergent response of leg press strength and leg extension strength to weight loss between groups further supports the notion that n-3PUFA supplementation enhanced neural adaptations. Although we observed an improvement in leg extension 1 RM with n-3PUFA supplementation, no difference in strength was observed for leg press 1 RM between groups. A possible explanation for this differential finding relates to the recruitment and activation of different muscle groups between exercises. Based on electromyography data, only the quadricep muscle group is activated during leg extension (33), whereas multiple muscle groups, including gastrocnemius, quadriceps and gluteus maximus are activated during the leg press (34). We report a regional decline in FFM in both left and right legs following weight loss in both groups (Supplementary Table). Expressed relative to total muscle mass activated during the exercise test, the decline in FFM in muscles activated during the leg extension was less than leg press. Hence, we speculate that the incorporation of DHA into neural tissue altered the interaction between the central nervous system and muscle tissue, potentially improving firing rate and recruitment of motor neurons during the 1 RM leg extension. Further research is warranted to substantiate this notion and to examine the influence of n-3PUFA supplementation on neuromuscular activity during leg press and leg extension exercises both under conditions of weight maintenance and weight loss.

The effective modification of body composition during weight loss with nutrition, and more specifically with manipulation of dietary protein, has been demonstrated previously in both clinical (35, 36) and athletic (18, 37) populations. In this regard, the protein content of an energy-restricted diet has been shown to modulate the magnitude of muscle loss during weight loss (2, 5). Recent evidence suggests an interactive effect of protein and n-3PUFA in the regulation of muscle protein metabolism. For example, Smith et al. (10) demonstrated a potentiated response of MPS to the intravenous infusion of amino acids following 8 weeks of n-3PUFA supplementation in young adults. Based on this observation, it is possible that a higher dietary protein intake was required in the present study for n-3PUFA supplementation to elicit a protective effect on lean body mass during weight loss. By design, in the present study dietary protein intake was reduced during the 2 week weight loss period in proportion to the energy deficit imposed. The basis for this methodological decision was to examine a proof-of-concept, i.e., testing the impact of n-3PUFA supplementation on body composition and muscle strength during weight loss rather than saturate any beneficial response with a high protein intake. Accordingly, during the energy restriction period, protein intake was 5% lower in FO and 7% lower in PLA compared to the habitual diet, equating to a reduction of 310 and 340 kcals, respectively. We also cannot discount the possibility that the combination of an energy deficit and reduction in dietary protein intake may have potentiated the decline in lean body mass during weight loss in both groups, thus countering any potential enhancement of n-3PUFA on MPS. Consistent with this notion, a negative nitrogen balance was reported for at least 10 days during a period of reduced protein intake (38). This negative nitrogen balance is indicative of a net loss of protein at the whole-body level. Conversely, increasing the protein content of the diet is known to increase nitrogen balance during energy restriction (37). The measurement of nitrogen balance was beyond the scope of the present study. However, based on previous work (39), we speculate that participants in both supplement conditions were in negative nitrogen balance. Future research in athletes is warranted to examine the influence of n-3PUFA supplementation on changes in body composition and muscle performance during energy restriction within a more practical situation when combined with a protein intake that meets recently published guidelines (4).

Although there are many strengths to the present study, including dietary control and the measurement of blood omega-3 concentrations, there also are limitations. Firstly, DEXA was the only measure of body composition and we acknowledge that DEXA measurements alone are not sufficient for the accurate assessment of muscle mass (40). Therefore, although all attempts were made to standardize the DEXA protocol (i.e., body positioning, hydration status), we cannot discount the possibility that our study incurred a type II statistical error with regards to examining the influence of FO supplementation on changes in body composition during short-term weight loss. Combining DEXA with measurements of bioelectrical impedance and air displacement plethysmography for calculating the four-compartmental model of body composition may provide more accurate results. Secondly, the participants that took part in this study were not a homogenous group. Although participants were resistance trained for at least 6 months, there were individuals and with different resistance abilities with different experience as well as being different types of athletes, i.e., power athletes or team-sports athletes. Thirdly, although we were able to fully control diet, we could only reliably control diet during the energy restriction phase for 2 weeks. A longer period of energy restriction may have allowed for further differences between the groups to have been observed.

To conclude, dietary supplementation with 4 g/d of n-3PUFA may maintain, or even improve, leg extension 1 RM strength following 2 weeks of energy restriction. However, this strength adaptation was not mediated by the increased preservation of lean body mass during diet-induced weight loss. The practical application of these preliminary data remains unclear but implies a potential role for n-3PUFA supplementation in improving muscle performance during weight loss in athletic populations. However, follow-up mechanistic work is warranted to establish the influence of n-3PUFA supplementation on changes in body composition, muscle performance and neuromuscular function during more prolonged periods of energy restriction in athletic populations.

## Data Availability

The raw data supporting the conclusions of this manuscript will be made available by the authors, without undue reservation, to any qualified researcher.

## Ethics Statement

All participants were informed of the purpose of the study, the experimental procedures, and all the potential risks involved. All subjects gave written informed consent in accordance with the Declaration of Helsinki. The protocol was approved by the West of Scotland Research Ethics Service.

## Author Contributions

The study was designed by JP, NB, DH, SM, SG, KT, and OW. Data were collected by JP, NB, and NR-S. Analyzed by JP, NB, EM, and JD. Data interpretation and manuscript preparation were undertaken by JP, NB, NR-S, DH, SG, KT, and OW.

### Conflict of Interest Statement

The authors declare that the research was conducted in the absence of any commercial or financial relationships that could be construed as a potential conflict of interest.
