# Supplementary material for: Influence of Fish Oil-Derived n-3 Fatty Acid Supplementation on Changes in Body Composition and Muscle Strength During Short-Term Weight Loss in Resistance-Trained Men
Source: Front Nutr. 2019 Jul 16;6:102. doi: 10.3389/fnut.2019.00102 (PMC6652803; doi:10.3389/fnut.2019.00102)
Supplement: Supplementary file 1 [file Table_1.DOCX]

**Table 1** – Regional differences in total body mass, lean body mass and fat mass at baseline (week 1), week 4 (100% diet) and week 6 (60% diet).

|  | **FO** | **CON** | **FO** | **CON** | **FO** | **CON** | ***P* (Group)** | ***P* (Time)** | ***P* (Group × Time)** |
| --- | --- | --- | --- | --- | --- | --- | --- | --- | --- |
| **Whole Body** |  |  |  |  |  |  |  |  |  |
| Mass | 88.45 ± 5.62 | 78.88 ± 3.51 | 88.71 ± 5.56 | 78.22 ± 3.46 | 85.62 ± 5.53 | 76 ± 3.39 | 0.169 | **<0.001** | 0.926 |
| Lean Mass | 67.48 ± 3.74 | 61.71 ± 2.91 | 67.45 ± 3.72 | 60.91 ± 2.79 | 66.01 ± 3.77 | 60.06 ± 2.79 | 0.21 | **<0.001** | 0.718 |
| Fat Mass | 17.48 ± 2.49 | 13.93 ± 2 | 17.96 ± 2.56 | 14.03 ± 1.95 | 16.16 ± 2.4 | 12.69 ± 1.99 | 0.276 | **<0.001** | 0.861 |
| **Limbs** |  |  |  |  |  |  |  |  |  |
| Mass | 42.55 ± 2.99 | 38.13 ± 1.7 | 42.59 ± 2.97 | 37.93 ± 1.62 | 41.4 ± 2.83 | 36.67 ± 1.69 | 0.212 | **<0.001** | 0.861 |
| Lean Mass | 33.17 ± 2.27 | 29.91 ± 1.53 | 33.01 ± 2.27 | 29.7 ± 1.45 | 32.3 ± 2.17 | 28.93 ± 1.49 | 0.233 | **<0.001** | 0.683 |
| Fat Mass | 7.55 ± 0.89 | 6.46 ± 0.77 | 7.72 ± 0.9 | 6.48 ± 0.78 | 7.25 ± 0.88 | 6.01 ± 0.82 | 0.329 | **<0.001** | 0.364 |
| **Trunk** |  |  |  |  |  |  |  |  |  |
| Mass | 40.71 ± 2.55 | 36.12 ± 1.68 | 40.85 ± 2.49 | 35.88 ± 1.69 | 39.08 ± 2.59 | 34.8 ± 1.55 | 0.165 | **<0.001** | 0.459 |
| Lean Mass | 30.65 ± 1.41 | 28.35 ± 1.35 | 30.69 ± 1.39 | 28.26 ± 1.32 | 30.15 ± 1.49 | 27.93 ± 1.28 | 0.248 | **0.006** | 0.781 |
| Fat Mass | 8.98 ± 1.6 | 6.76 ± 1.19 | 9.08 ± 1.64 | 6.61 ± 1.2 | 7.87 ± 1.52 | 5.87 ± 1.15 | 0.273 | **<0.001** | 0.535 |
| **Right Side** |  |  |  |  |  |  |  |  |  |
| Mass | 44.1 ± 2.75 | 39.73 ± 1.74 | 44.15 ± 2.68 | 39.42 ± 1.72 | 42.52 ± 2.64 | 38.33 ± 1.78 | 0.543 | **<0.001** | 0.206 |
| Lean Mass | 33.51 ± 1.84 | 30.9 ± 1.44 | 33.42 ± 1.78 | 30.66 ± 1.4 | 32.72 ± 1.78 | 30.19 ± 1.45 | 0.268 | **<0.001** | 0.667 |
| Fat Mass | 8.8 ± 1.21 | 7.16 ± 0.96 | 8.95 ± 1.26 | 7.09 ± 0.98 | 8.04 ± 1.18 | 6.45 ± 1 | 0.29 | **<0.001** | 0.789 |
| **Left Side** |  |  |  |  |  |  |  |  |  |
| Mass | 44.38 ± 2.87 | 39.61 ± 1.71 | 44.54 ± 2.89 | 39.48 ± 1.65 | 43.12 ± 2.89 | 38.25 ± 1.5 | 0.176 | **<0.001** | 0.821 |
| Lean Mass | 33.96 ± 1.91 | 30.98 ± 1.47 | 33.99 ± 1.95 | 30.9 ± 1.42 | 33.35 ± 1.97 | 30.26 ± 1.33 | 0.219 | **0.002** | 0.775 |
| Fat Mass | 8.68 ± 1.29 | 7 ± 0.97 | 8.81 ± 1.29 | 6.92 ± 0.98 | 8.02 ± 1.23 | 6.34 ± 0.97 | 0.287 | **<0.001** | 0.988 |
| **Right Arm** |  |  |  |  |  |  |  |  |  |
| Mass | 5.85 ± 0.4 | 5.19 ± 0.27 | 5.8 ± 0.37 | 5.14 ± 0.26 | 5.6 ± 0.31 | 5.04 ± 0.28 | 0.2 | 0.34 | 0.578 |
| Lean Mass | 4.76 ± 0.35 | 4.23 ± 0.26 | 4.71 ± 0.33 | 4.18 ± 0.24 | 4.55 ± 0.29 | 4.18 ± 0.26 | 0.254 | 0.08 | 0.288 |
| Fat Mass | 0.84 ± 0.08 | 0.71 ± 0.1 | 0.83 ± 0.08 | 0.72 ± 0.1 | 0.79 ± 0.08 | 0.66 ± 0.1 | 0.344 | **0.001** | 0.907 |
| **Right Leg** |  |  |  |  |  |  |  |  |  |
| Mass | 15.35 ± 1.05 | 13.91 ± 0.57 | 15.47 ± 1.1 | 13.85 ± 0.57 | 15.06 ± 1.08 | 13.4 ± 0.6 | 0.236 | **0.001** | 0.281 |
| Lean Mass | 11.74 ± 0.76 | 10.72 ± 0.49 | 11.72 ± 0.8 | 10.63 ± 0.49 | 11.53 ± 0.78 | 10.39 ± 0.51 | 0.253 | **0.003** | 0.41 |
| Fat Mass | 2.96 ± 0.36 | 2.55 ± 0.29 | 3.06 ± 0.37 | 2.57 ± 0.29 | 2.85 ± 0.36 | 2.38 ± 0.32 | 0.342 | **0.004** | 0.402 |
| **Left Arm** |  |  |  |  |  |  |  |  |  |
| Mass | 5.84 ± 0.47 | 5.22 ± 0.28 | 5.8 ± 0.44 | 5.2 ± 0.25 | 5.64 ± 0.46 | 4.97 ± 0.25 | 0.272 | **<0.001** | 0.575 |
| Lean Mass | 4.76 ± 0.41 | 4.26 ± 0.26 | 4.71 ± 0.39 | 4.25 ± 0.23 | 4.61 ± 0.39 | 4.08 ± 0.23 | 0.3 | **0.001** | 0.761 |
| Fat Mass | 0.82 ± 0.1 | 0.71 ± 0.1 | 0.83 ± 0.1 | 0.71 ± 0.1 | 0.77 ± 0.1 | 0.65 ± 0.1 | 0.407 | **<0.001** | 0.717 |
| **Left Leg** |  |  |  |  |  |  |  |  |  |
| Mass | 15.51 ± 1.13 | 13.81 ± 0.62 | 15.54 ± 1.11 | 13.77 ± 0.6 | 15.14 ± 1.08 | 13.04 ± 0.59 | 0.191 | **<0.001** | 0.262 |
| Lean Mass | 11.92 ± 0.82 | 10.7 ± 0.55 | 11.86 ± 0.82 | 10.66 ± 0.52 | 11.61 ± 0.81 | 10.28 ± 0.51 | 0.212 | **<0.001** | 0.419 |
| Fat Mass | 2.93 ± 0.38 | 2.48 ± 0.3 | 3 ± 0.37 | 2.48 ± 0.3 | 2.84 ± 0.35 | 2.32 ± 0.31 | 0.308 | **0.003** | 0.372 |

All values are means ± SEM.
